# Risk of second primary cancer among women in the Kaiser Permanente Breast Cancer Survivors Cohort

**DOI:** 10.1186/s13058-023-01647-y

**Published:** 2023-05-03

**Authors:** Cody Ramin, Lene H. S. Veiga, Jacqueline B. Vo, Rochelle E. Curtis, Clara Bodelon, Erin J. Aiello Bowles, Diana S. M. Buist, Sheila Weinmann, Heather Spencer Feigelson, Gretchen L. Gierach, Amy Berrington de Gonzalez

**Affiliations:** 1 Division of Cancer Epidemiology and Genetics, National Cancer Institute, 9609 Medical Center Drive, Bethesda, MD USA; 2 Cancer Research Center for Health Equity, Cedars-Sinai Medical Center, Los Angeles, CA USA; 3 Kaiser Permanente Washington Health Research Institute, Kaiser Permanente Washington, Seattle, WA USA; 4 Bernard J. Tyson Kaiser Permanente School of Medicine, Pasadena, CA USA; 5 Kaiser Permanente Center for Health Research, Kaiser Permanente Northwest, Portland, OR USA; 6 Institute for Health Research, Kaiser Permanente, Denver, CO USA; 7 Division of Genetics and Epidemiology, ICR, London, UK

**Keywords:** Breast cancer, Cancer survivorship, Second cancers, Second non-breast cancers, Endocrine therapy, Radiotherapy, Chemotherapy

## Abstract

**Background:**

Breast cancer survivors are living longer due to early detection and advances in treatment and are at increased risk for second primary cancers. Comprehensive evaluation of second cancer risk among patients treated in recent decades is lacking.

**Methods:**

We identified 16,004 females diagnosed with a first primary stage I-III breast cancer between 1990 and 2016 (followed through 2017) and survived ≥ 1 year at Kaiser Permanente (KP) Colorado, Northwest, and Washington. Second cancer was defined as an invasive primary cancer diagnosed ≥ 12 months after the first primary breast cancer. Second cancer risk was evaluated for all cancers (excluding ipsilateral breast cancer) using standardized incidence ratios (SIRs), and a competing risk approach for cumulative incidence and hazard ratios (HRs) adjusted for KP center, treatment, age, and year of first cancer diagnosis.

**Results:**

Over a median follow-up of 6.2 years, 1,562 women developed second cancer. Breast cancer survivors had a 70% higher risk of any cancer (95%CI = 1.62–1.79) and 45% higher risk of non-breast cancer (95%CI = 1.37–1.54) compared with the general population. SIRs were highest for malignancies of the peritoneum (SIR = 3.44, 95%CI = 1.65–6.33), soft tissue (SIR = 3.32, 95%CI = 2.51–4.30), contralateral breast (SIR = 3.10, 95%CI = 2.82–3.40), and acute myeloid leukemia (SIR = 2.11, 95%CI = 1.18–3.48)/myelodysplastic syndrome (SIR = 3.25, 95%CI = 1.89–5.20). Women also had elevated risks for oral, colon, pancreas, lung, and uterine corpus cancer, melanoma, and non-Hodgkin lymphoma (SIR range = 1.31–1.97). Radiotherapy was associated with increased risk for all second cancers (HR = 1.13, 95%CI = 1.01–1.25) and soft tissue sarcoma (HR = 2.36, 95%CI = 1.17–4.78), chemotherapy with decreased risk for all second cancers (HR = 0.87, 95%CI = 0.78–0.98) and increased myelodysplastic syndrome risk (HR = 3.01, 95%CI = 1.01–8.94), and endocrine therapy with lower contralateral breast cancer risk (HR = 0.48, 95%CI = 0.38–0.60). Approximately 1 in 9 women who survived ≥ 1 year developed second cancer, 1 in 13 developed second non-breast cancer, and 1 in 30 developed contralateral breast cancer by 10 years. Trends in cumulative incidence declined for contralateral breast cancer but not for second non-breast cancers.

**Conclusions:**

Elevated risks of second cancer among breast cancer survivors treated in recent decades suggests that heightened surveillance is warranted and continued efforts to reduce second cancers are needed.

**Supplementary Information:**

The online version contains supplementary material available at 10.1186/s13058-023-01647-y.

## Background

There are nearly 4 million breast cancer survivors in the US, and this number is increasing due to an aging population and improvements in breast cancer survival [1]. During recent decades, advances in screening and treatment have contributed to a 5-year survival rate that has reached 90% for all stages and 99% for localized stage [1–4]. Although breast cancer survivors are living longer, they have substantially increased risk of developing and dying from a second cancer [5–19]. Previous studies have established that second cancers can develop as a late effect of breast cancer treatment [20]. An increased risk of endometrial cancer has been observed after tamoxifen therapy [7, 15], leukemia and myelodysplastic syndrome after either chemotherapy [7, 21–24] or radiotherapy [15, 25, 26], and soft tissue sarcomas, lung, breast, and esophageal cancer after radiotherapy [7, 15, 27, 28]. Endocrine therapy has also been shown to significantly reduce the risk of second breast cancer [29–33]. Importantly, breast cancer treatment has changed considerably over the last several decades with shifts in chemotherapy regimens, improvements in radiotherapy techniques, widespread use of endocrine therapies, and increases in contralateral prophylactic mastectomies [3, 34]. However, prior studies evaluating second cancer risk have been primarily conducted among registry-based studies and limited to women diagnosed and treated in the mid-2000s or earlier [5–13]. Therefore, second cancer risk after significant advances and changes in breast cancer treatment and within an integrated health care delivery system have not been well described.

Here we evaluated second cancer risk among a large retrospective cohort of breast cancer survivors diagnosed between 1990 and 2016 (followed through 2017) within an integrated US health care delivery system with long-term follow-up and comprehensive treatment data. Our study utilizes systematically ascertained data on radiotherapy, chemotherapy, and endocrine therapy to examine second cancer risk that cannot be addressed with Surveillance, Epidemiology, and End Results (SEER) registry data due to the known under-ascertainment of treatment and availability of initial treatment only [35]. A comprehensive assessment of second cancer risk by age, tumor, and treatment characteristics of the first breast cancer among women diagnosed and treated within an integrated health care delivery system could inform contemporary strategies for clinical surveillance and efforts to reduce second cancer risk among breast cancer survivors.

## Methods

### Study population

The Kaiser Permanente (KP) Breast Cancer Survivors Cohort is a retrospective cohort of women diagnosed with a first primary unilateral breast cancer at three KP sites: Northwest (*n* = 4,658 between 1990 and 2008), Colorado (*n* = 5,512 between 1994 and 2014), or Washington (*n* = 8,242 between 1990 and 2016). Eligible women were KP members who survived and remained at risk for second cancer for at least 1 year. We excluded women diagnosed with a first breast cancer at age < 20 years (*n* = 1) or ≥ 85 years (*n* = 563), in situ (*n* = 1,478) or metastatic disease (*n* = 204), unknown stage (*n* = 72), and those not treated with surgery (*n* = 90) (Additional file 1: Fig. S1). This left 16,004 women in the analytic study population. This study was approved by the National Institutes of Health Institutional Review Board (IRB) and by the IRBs of KP Northwest, Colorado, and Washington.

### Covariate and cancer ascertainment

Patient information was extracted from electronic medical record (EMR) databases, including date of birth, race, ethnicity, body mass index (BMI), and smoking status. BMI was calculated from height and weight measurements within 1 year before to 1 year after first breast cancer diagnosis and supplemented with chart review to fill in missing data. Smoking status was obtained at first breast cancer diagnosis through 1 year after diagnosis from social history records of EMRs and supplemented using ICD-9 (305.1, V15.82, V65.42), ICD-10 (F17.200, Z87.891, Z72.0), and procedure codes (4000, 200162, 99406, 99407, S9075). Cancer diagnoses and tumor characteristics were obtained from cancer registries (KP tumor registries for KP Colorado and Northwest, and SEER registry for KP Washington). Breast cancer tumor characteristics included stage, laterality, histologic type, estrogen receptor (ER), progesterone receptor (PR), and human epidermal growth factor receptor 2 (HER2) status. Chemotherapy and endocrine therapy data were obtained from KP electronic pharmacy records and included information on specific drug names and dispensing dates. Data on chemotherapy were supplemented with information from tumor registries to capture patients that could have been treated outside of KP (< 4%). Endocrine therapy and chemotherapy were evaluated for the entire follow-up period. Radiotherapy was obtained from KP tumor registries and included the first course of therapy only.

### Second cancer outcomes

Second cancer was defined as an invasive primary cancer diagnosed ≥ 12 months after the first primary breast cancer diagnosis. Second cancers were primarily identified according to the ICD-O-3 site and morphology codes (Additional file 1: Table S1) [36]. Bone and soft-tissue sarcomas were defined based on an extended classification of the International Classification of Childhood Cancers, third edition (ICCC-3) [37, 38]. Results are presented for 1) all second cancers (excluding ipsilateral breast cancer), 2) all second non-breast cancers, and 3) site-specific second cancers with ≥ 10 events unless specified a priori as a site of interest (e.g., esophageal cancer). Ipsilateral breast cancers (*n* = 144) were censored at date of diagnosis to reduce potential misclassification of a recurrence as a second cancer. Analyses for contralateral breast cancer excluded women who underwent a contralateral prophylactic mastectomy (*n* = 1,042). Since myelodysplastic syndromes were not ascertained in SEER until 2001, analyses for these events were restricted to 2001–2017.

### Statistical analysis

Women were followed beginning 12 months after their initial breast cancer diagnosis until the first of the following: second cancer diagnosis, death, health plan exit, or end of follow-up (Additional file 1: Fig. S1). Cumulative incidence was calculated for the 10 most common second cancers and by decade of first breast cancer diagnosis using nonparametric methods accounting for competing events [39]. We calculated standardized incidence ratios (SIRs; observed/expected) and exact 95% confidence intervals (CIs) to compare incident cancers among breast cancer patients to expected first cancers in the general population. To calculate the expected number of cancers, we used the nine US SEER registries as the reference population and obtained age-, race-, and calendar-time specific first cancer incidence rates multiplied by the person-time in each stratum. SIRs for all second cancers, second non-breast cancers, and site-specific second cancers were estimated overall and stratified by first breast cancer characteristics and treatment. Results were stratified by age < 55/ ≥ 55 years at first breast cancer (proxy for menopausal status) and restricted to age < 45 years to examine risk among younger women (based on the distribution of the study population). To examine the potential effect of medical surveillance bias, we also examined SIRs by time after initial diagnosis (i.e., latency). Results with < 5 events were omitted in stratified analyses for site-specific second cancers. To compare SIRs in stratified analyses, we used Poisson regression with the observed number of cases as the outcome, the log of the expected number of cases as the offset, and the stratified factor as a covariate in the model [40, 41]. P-values for heterogeneity were based on the likelihood ratio statistic comparing model fit with and without the stratified factor.

To further examine the association between treatment and second cancer risk, we used Fine and Gray regression with time since index date as the time scale to estimate subdistribution hazard ratios (HRs) accounting for competing events [42], and adjusting for radiotherapy, chemotherapy, and endocrine therapy (separate binary yes/no variables). Multivariable models additionally adjusted for age at first breast cancer diagnosis (continuous), year of first breast cancer diagnosis (5-year categories), and KP center. Adjustment for BMI (< 25, 25- < 30, ≥ 30 kg/m^2^), smoking (ever, never), and clinicopathological characteristics of the first breast cancer, including stage (I, II, III), histology (ductal, lobular, mixed, other), and ER/PR status did not change the results; therefore, the more parsimonious model was used. Models examining endocrine therapy were restricted to first ER-positive breast cancers. HRs estimated with Cox proportional hazard regression are reported in the supplement. Due to a potentially longer latency period between treatment and second cancer risk, we also examined associations restricted to 5-year survivors.

All *p*-values < 0.05 were considered statistically significant and tests were two-sided. Analyses were performed using SEER*Stat 8.3.9 and Stata 16 (College Station, TX).

## Results

The mean age at first breast cancer diagnosis was 60.7 years (standard deviation, 12.0) and the mean year of diagnosis was 2003 (standard deviation, 6.9) (Table 1). First breast cancers were predominately stage I (58.4%), ductal (76.7%), and ER-positive (79.6%). Women primarily underwent breast conserving surgery (61.1%) and received radiotherapy (66.5%) and/or endocrine therapy (70.0%). During a median follow-up of 6.2 years, 1,562 women developed a second cancer. Women who developed second cancer were less likely to have received chemotherapy or endocrine therapy compared with women who did not develop second cancer.

**Table 1:** Selected patient and clinical characteristics among 16,004 women diagnosed with a first primary unilateral invasive breast cancer at three Kaiser Permanente sites, 1990–2016 and followed through 2017

		Second cancer case status		
Characteristics of the first breast cancer	Total (N = 16,004)	Second cancer cases (*n* = 1,562)	Second non-breast cancer cases (*n* = 1,112)	Non-cases (*n* = 14,298)^a^
	n (%)	n (%)	n (%)	n (%)
Year of diagnosis, mean (SD)	2003.0 (6.9)	1999.3 (5.9)	1999.5 (6.0)	2003.4 (6.8)
Year of diagnosis^b^	–			
1990–1994	2035 (12.7)	380 (24.3)	268 (24.1)	1623 (11.4)
1995–1999	3426 (21.4)	466 (29.8)	314 (28.2)	2899 (20.3)
2000–2004	3696 (23.1)	394 (25.2)	281 (25.3)	3267 (22.9)
2005–2009	3541 (22.1)	235 (15.0)	182 (16.4)	3293 (23.0)
2010–2016	3306 (20.7)	87 (5.6)	67 (6.0)	3216 (22.5)
Age at diagnosis, years, mean (SD)	60.7 (12.0)	63.4 (11.2)	64.6 (10.8)	60.5 (12.0)
Age at diagnosis, years				
20–39	630 (3.9)	41 (2.6)	18 (1.6)	579 (4.1)
40–49	2635 (16.5)	174 (11.1)	103 (9.3)	2424 (17.0)
50–59	4325 (27.0)	341 (21.8)	229 (20.6)	3944 (27.6)
60–69	4452 (27.8)	523 (33.5)	375 (33.7)	3895 (27.2)
70–79	3117 (19.5)	394 (25.2)	315 (28.3)	2705 (18.9)
80–84	845 (5.3)	89 (5.7)	72 (6.5)	751 (5.3)
Race				
White	14,691 (91.8)	1468 (94.0)	1055 (94.9)	13,091 (91.6)
Black	458 (2.9)	38 (2.4)	21 (1.9)	416 (2.9)
American Indian/Alaskan Native	91 (0.6)	6 (0.4)	5 (0.5)	82 (0.6)
Asian/Pacific Islander	602 (3.8)	42 (2.7)	27 (2.4)	556 (3.9)
Other	49 (0.3)	4 (0.3)	3 (0.3)	45 (0.3)
Unknown	113 (0.7)	4 (0.3)	1 (0.1)	108 (0.8)
Ethnicity^*c*^				
Non-Hispanic	10,902 (93.8)	1031 (94.7)	749 (95.4)	9767 (93.8)
Hispanic	660 (5.7)	58 (5.3)	36 (4.6)	592 (5.7)
Unknown	58 (0.5)	0 (0)	0 (0)	58 (0.6)
Body mass index, kg/m^2^, mean (SD)	28.7 (6.7)	29.0 (6.8)	28.8 (6.7)	28.7 (6.6)
Ever tobacco use	5182 (32.4)	454 (29.1)	333 (30.0)	4698 (32.9)
Stage				
I	9348 (58.4)	998 (63.9)	715 (64.3)	8245 (57.7)
II	5350 (33.4)	457 (29.3)	322 (29.0)	4857 (34.0)
III	1306 (8.2)	107 (6.9)	75 (6.7)	1196 (8.4)
Histology				
Ductal	12,271 (76.7)	1197 (76.6)	847 (76.2)	10,965 (76.7)
Lobular	1414 (8.8)	138 (8.8)	105 (9.4)	1268 (8.9)
Mixed	1041 (6.5)	89 (5.7)	60 (5.4)	942 (6.6)
Other	1278 (8.0)	138 (8.8)	100 (9.0)	1123 (7.9)
ER status				
Negative	2674 (16.7)	255 (16.3)	170 (15.3)	2401 (16.8)
Positive	12,746 (79.6)	1237 (79.2)	894 (80.4)	11,397 (79.7)
Unknown/borderline	584 (3.6)	70 (4.5)	48 (4.3)	500 (3.5)
PR status				
Negative	4341 (27.1)	418 (26.8)	300 (27.0)	3889 (27.2)
Positive	10,972 (68.6)	1055 (67.5)	753 (67.7)	9823 (68.7)
Unknown/borderline	691 (4.3)	89 (5.7)	59 (5.3)	586 (4.1)
HER2 status^*d*^				
Negative	2792 (84.5)	76 (87.4)	58 (86.6)	2714 (84.4)
Positive	412 (12.5)	6 (6.9)	5 (7.5)	406 (12.6)
Unknown/borderline	102 (3.1)	5 (5.8)	4 (6.0)	96 (3.0)
Surgery type				
Lumpectomy, partial mastectomy	9772 (61.1)	994 (63.6)	694 (62.4)	8634 (60.4)
Mastectomy	6232 (38.9)	568 (36.4)	418 (37.6)	5664 (39.6)
Received radiotherapy				
No	5302 (33.1)	507 (32.5)	381 (34.3)	4783 (33.5)
Yes	10,638 (66.5)	1054 (67.5)	731 (65.7)	9452 (66.1)
Unknown	64 (0.4)	1 (0.1)	0 (0)	63 (0.4)
Received chemotherapy				
No	9218 (57.6)	1028 (65.8)	753 (67.7)	8097 (56.6)
Yes	6786 (42.4)	534 (34.2)	359 (32.3)	6201 (43.4)
Received endocrine therapy				
No	4805 (30.0)	563 (36.0)	363 (32.6)	4177 (29.2)
Yes	11,199 (70.0)	999 (64.0)	749 (67.4)	10,121 (70.8)
Tamoxifen only	5147 (46.0)	640 (64.1)	469 (62.6)	4448 (44.0)
AIs only	3012 (26.9)	154 (15.4)	123 (16.4)	2850 (28.2)
Tamoxifen + AIs	2611 (23.3)	167 (16.7)	127 (17.0)	2435 (24.1)
Other/unknown	429 (3.8)	38 (3.8)	30 (4.0)	388 (3.8)

Abbreviations *ER* Estrogen receptor, *PR* Progesterone receptor, *HER2* Human epidermal growth factor receptor 2, *AI* Aromatase inhibitor

^a^144 women were diagnosed with a second ipsilateral breast cancer and censored at the date of diagnosis. These women were not included in the distribution for cases and non-cases

^b^Women were diagnosed with stage I-III breast cancer between 1990–2016 and followed through 2017

^c^Ethnicity was available for Kaiser Permanente Colorado and Washington

^d^HER2 status was restricted to women diagnosed with a first breast cancer in 2010–2016 and followed through 2017

**Fig. 1:**
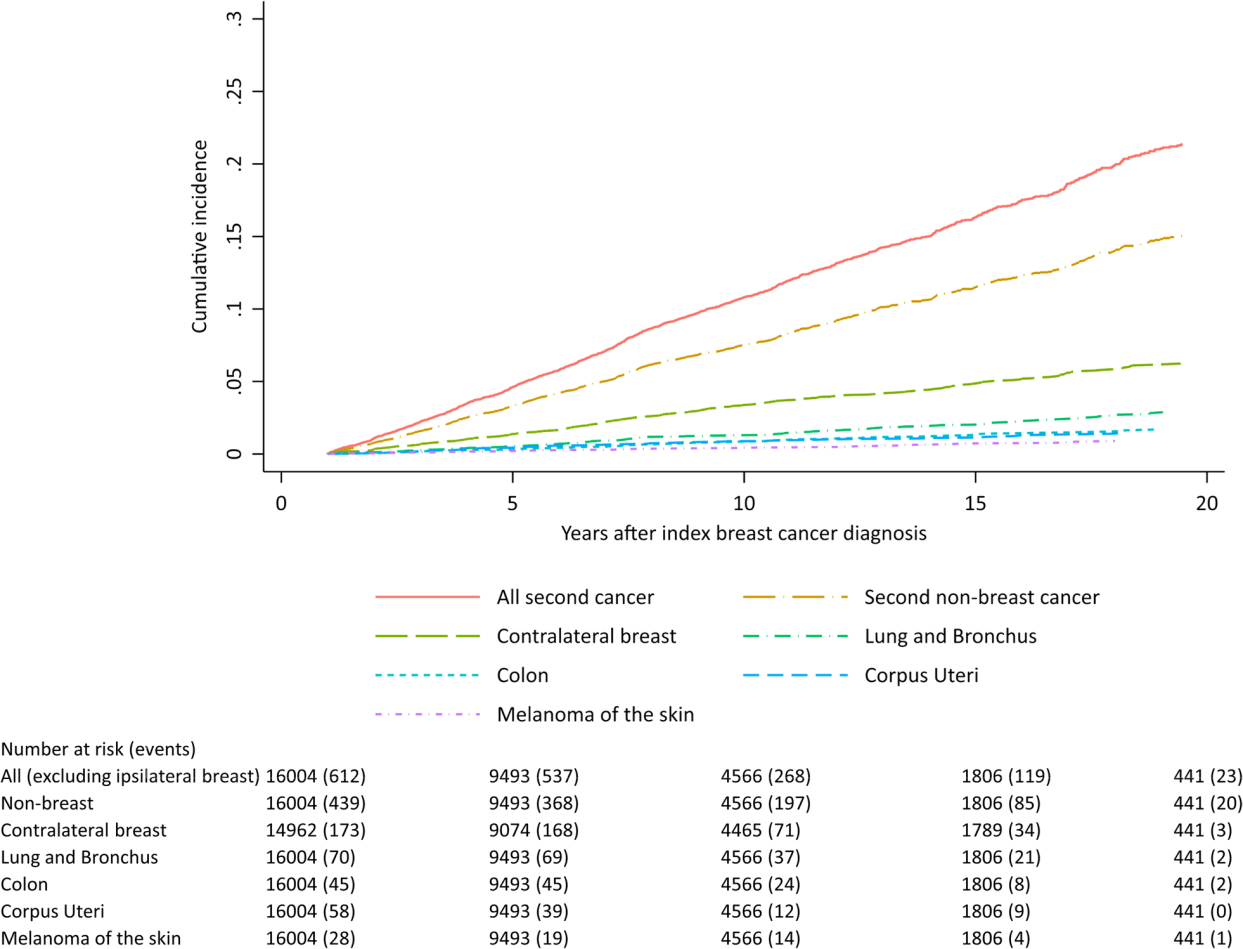
Cumulative incidence of second primary cancer among 16,004 women diagnosed with a first primary unilateral invasive breast cancer between 1990 to 2016 and followed through 2017

The 10-year cumulative incidence was 10.8% for all second cancers, 7.5% for non-breast cancer, and 3.4% for contralateral breast cancer (Fig. 1, Additional file 1: Table S2). Cumulative incidence for lung, colon, uterine corpus, melanoma, soft tissue sarcoma, and leukemia was low (≤ 1% at 10 years). The cumulative incidence of contralateral breast cancer declined by year of first breast cancer diagnosis, but no decline was observed for other second cancers (Table 2, Additional file 1: Fig. S2).

**Table 2:** Cumulative incidence for second primary cancer according to year of first breast cancer diagnosis among 16,004 women diagnosed with a first primary unilateral invasive breast cancer^a^

Year of first breast cancer diagnosis	5 years	10 years
	% (95% CI)	% (95% CI)
*All second cancers*		
1990- < 2000	4.77 (4.21–5.38)	10.97 (10.09–11.90)
2000- < 2010	4.69 (4.19–5.24)	10.79 (9.92–11.70)
2010 +	4.19 (3.27–5.38)	–^b^
*All second non-breast cancers*		
1990- < 2000	3.18 (2.72–3.68)	7.14 (6.43–7.91)
2000- < 2010	3.49 (3.06–3.97)	7.87 (7.13–8.66)
2010 +	3.36 (2.54–4.35)	–^b^
*Contralateral breast*^*c*^		
1990- < 2000	1.61 (1.29–1.99)	3.82 (3.30–4.41)
2000- < 2010	1.28 (1.02–1.60)	3.05 (2.57–3.58)
2010 +	0.98 (0.56–1.61)	–^b^

^a^Cumulative incidence for all second primary cancers, second non-breast cancers, and contralateral breast cancer were estimated accounting for the competing risk of death and other invasive cancers (contralateral breast cancer only)

^b^Follow-up time was not sufficient to report cumulative incidence at 10-years

^c^Women with bilateral mastectomies were excluded (*n* = 1,042)

**Fig. 2:**
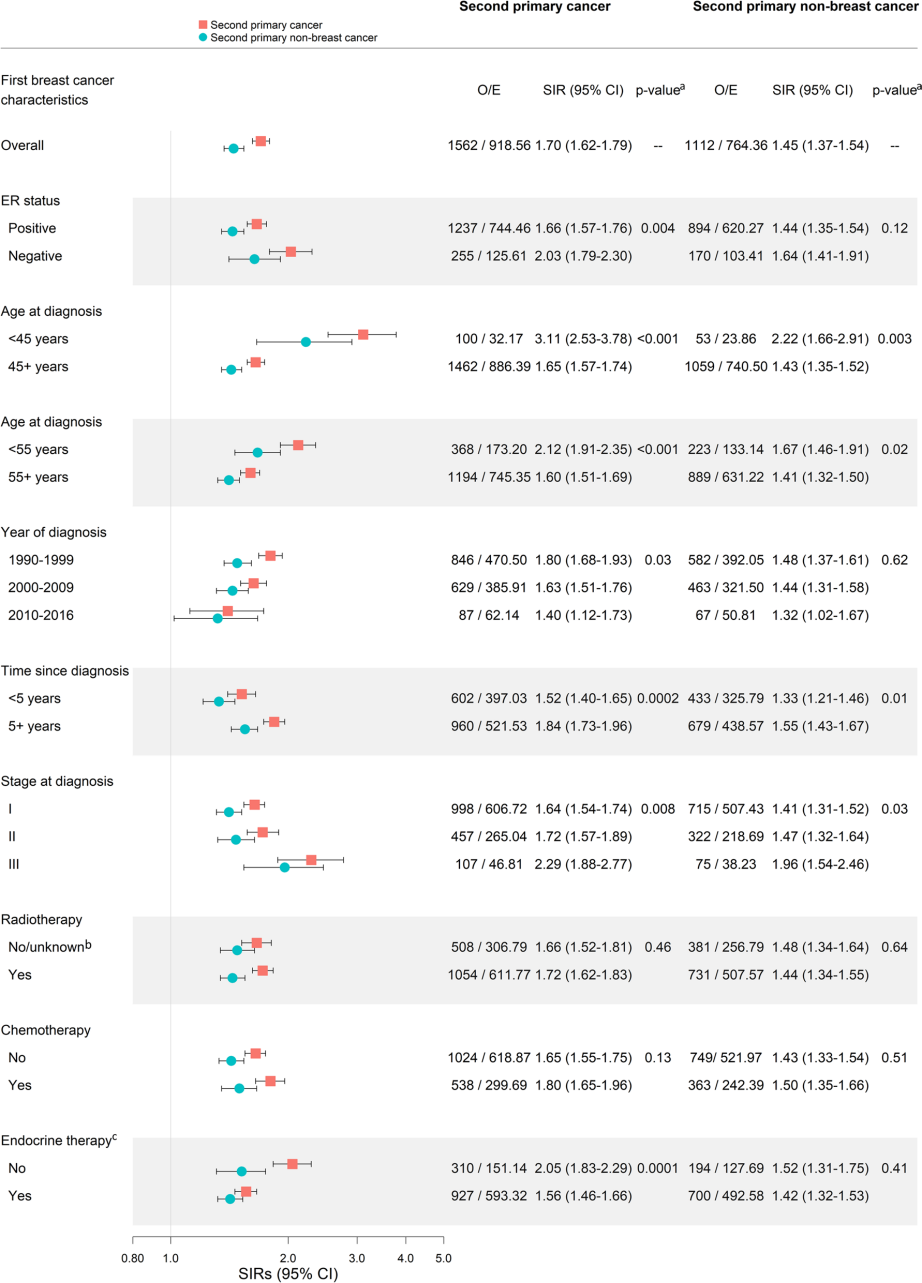
Standardized incidence ratios (SIRs) and 95% confidence intervals (CIs) for second primary cancer according to characteristics of the first breast cancer among 16,004 women diagnosed with a first primary unilateral invasive breast cancer between 1990 to 2016 and followed through 2017. *Abbreviations*: SIRs–Standardized incidence ratios, CI–Confidence interval, O–Observed, E–Expected, ER–Estrogen receptor. *Note*: Second primary cancer excludes second ipsilateral breast cancer. ^a^P-values to test for heterogeneity between SIRs. ^b^No/unknown receipt of radiotherapy is combined due to potential under ascertainment of radiotherapy in registry data (no radiotherapy: *n* = 5,302; unknown radiotherapy: *n* = 64). ^c^Restricted to women diagnosed with a first ER-positive breast cancer (*n* = 12,746)

Breast cancer survivors had significantly higher risk for all second cancers and non-breast cancers compared with the general population (SIR = 1.70, 95%CI = 1.62–1.79; SIR = 1.45, 95%CI = 1.37–1.54, respectively) (Fig. 2). Second cancer risk significantly varied by first breast cancer characteristics and treatment, including age, year, latency, ER status, stage, and endocrine therapy (*P*_heterogeneity_ < 0.05). SIRs for second cancer were particularly elevated (SIRs ≥ 2.00) for women diagnosed with a first breast cancer at a younger age, and after a stage III breast cancer, ER-negative breast cancer, or ER-positive breast cancer without endocrine therapy. Although second cancer risk remained elevated regardless of latency, risk was higher 5 + years after diagnosis (< 5 years: SIR = 1.52, 95%CI = 1.40–1.65; 5 + years: SIR = 1.84, 95%CI = 1.73–1.96; *P*_heterogeneity_ < 0.0002). SIRs for second non-breast cancer were attenuated compared to SIRs for all second cancers, particularly for ER status, year of diagnosis, and receipt of endocrine therapy, but otherwise patterns of risk remained similar.

**Fig. 3:**
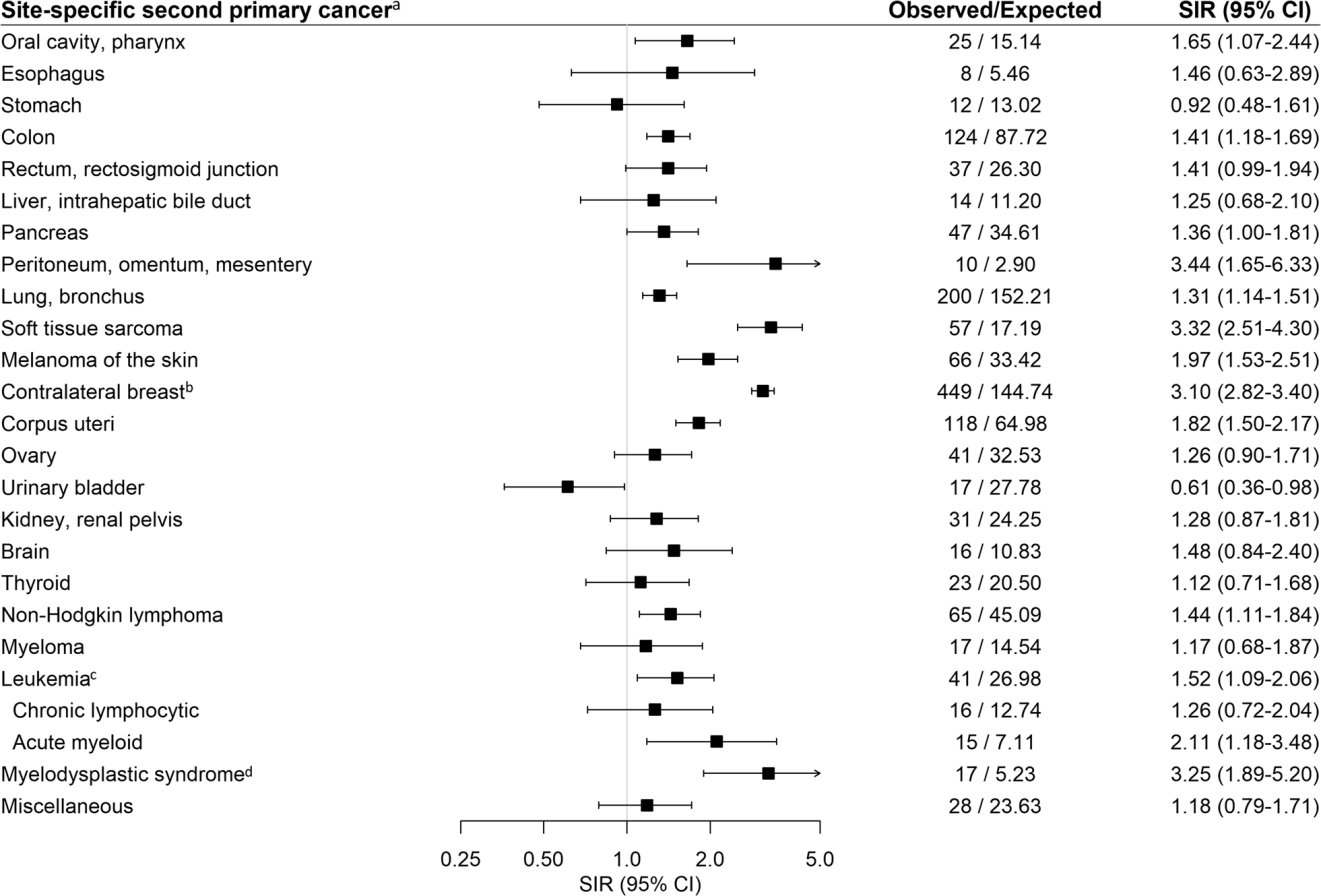
Standardized incidence ratios (SIRs) and 95% confidence intervals (CIs) for selected site-specific second primary cancers among 16,004 women diagnosed with a first primary unilateral invasive breast cancer between 1990 to 2016 and followed through 2017. *Abbreviations*: SIRs–Standardized incidence ratios, CIs–Confidence intervals, O–Observed, E–Expected. ^a^Cancer sites with ≥ 10 cases are presented unless specified a priori as a site of interest. ^b^Women with bilateral mastectomies were excluded (*n* = 1,042). ^c^Leukemia subtypes not presented include acute lymphocytic leukemia (*n* = 2), chronic myeloid leukemia (*n* = 6), other leukemia (*n* = 2). ^d^Analyses for myelodysplastic syndrome are restricted to 2001–2017 since SEER did not systematically ascertain this outcome prior to this date

Site-specific second cancer risk was highest for contralateral breast cancer (SIR = 3.10, 95%CI = 2.82–3.40), soft tissue sarcoma (SIR = 3.32, 95%CI = 2.51–4.30), peritoneal cancer (SIR = 3.44, 95%CI = 1.65–6.33), and myelodysplastic syndrome (SIR = 3.25, 95%CI = 1.89–5.20) (Fig. 3). Significantly elevated risk was also observed for malignancies of the oral cavity and pharynx (SIR = 1.65, 95%CI = 1.07–2.44), colon (SIR = 1.41, 95%CI = 1.18–1.69), pancreas (SIR = 1.36, 95%CI = 1.00–1.81), lung and bronchus (SIR = 1.31, 95%CI = 1.14–1.51), uterine corpus (SIR = 1.82, 95%CI = 1.50–2.17), melanoma (SIR = 1.97, 95%CI = 1.53–2.51), non-Hodgkin lymphoma (SIR = 1.44, 95%CI = 1.11–1.84), and acute myeloid leukemia (SIR = 2.11, 95%CI = 1.18–3.48). Lower risk was observed for bladder cancer (SIR = 0.61, 95%CI = 0.36–0.98).

SIRs for site-specific second cancers varied by first breast cancer characteristics and treatment (Additional file 1: Tables S3–7). SIRs were particularly elevated after an ER-negative breast cancer and significantly differed by ER status for lung and bronchus, ovarian, and contralateral breast cancer (*P*_heterogeneity_ < 0.05) (Additional file 1: Table S3). For results stratified by age < 55/ ≥ 55 years, SIRs were highest among women aged < 55 years and for malignancies of the oral cavity and pharynx, contralateral breast, soft tissue sarcoma, and melanoma (SIRs range = 2.58–4.55), but significant heterogeneity was only observed for contralateral breast cancer and melanoma (*P*_heterogeneity_ < 0.05) (Additional file 1: Table S4). Among women aged < 45 years, risk was further elevated for soft tissue sarcoma (SIR = 11.06, 95%CI = 5.06–20.99) and contralateral breast cancer (SIR = 6.10, 95%CI = 4.48–8.11) and was significantly elevated for ovarian (SIR = 3.63, 95%CI = 1.18–8.46) and thyroid cancer (SIR = 3.04, 95%CI = 1.31–5.98). SIRs stratified by treatment are presented in the data supplement (Additional file 1: Table S5-7).

In multivariable adjusted models, radiotherapy was associated with an increased risk for all second cancers (HR = 1.13, 95%CI = 1.01–1.25) and soft tissue sarcoma (HR = 2.36, 95%CI = 1.17–4.78) (Table 3). Chemotherapy was associated with lower risk for all second cancers (HR = 0.87, 95%CI = 0.78–0.98) and increased risk for myelodysplastic syndrome (HR = 3.01, 95%CI = 1.01–8.94). ER-positive patients treated with endocrine therapy had a decreased risk for all second cancers (HR = 0.78, 95%CI = 0.68–0.89) and contralateral breast cancer (HR = 0.48, 95%CI = 0.38–0.60). Results remained similar overall when using Cox proportional hazards regression (Additional file 1: Table S8) and were slightly attenuated when restricted to 5-year survivors (Additional file 1: Table S9).

**Table 3:** Associations between breast cancer treatment and risk of developing second primary cancer among 16,004 women diagnosed with a first primary unilateral invasive breast cancer between 1990 to 2016 and followed through 2017^a,b^

	Age-adjusted HRs (95% CIs)^c^			Multivariable-adjusted HRs (95% CIs)^d^		
Site-specific second primary cancer	Radiotherapy	Chemotherapy	Endocrine therapy^e^	Radiotherapy	Chemotherapy	Endocrine therapy^e^
All second cancer	1.10 (0.99–1.22)	**0.86 (0.77–0.97)**	**0.74 (0.65–0.84)**	**1.13 (1.01–1.25)**	**0.87 (0.78–0.98)**	**0.78 (0.68–0.89)**
All second non-breast cancer	1.04 (0.92–1.17)	0.88 (0.77–1.01)	0.94 (0.80–1.11)	1.06 (0.93–1.20)	0.89 (0.77–1.02)	0.99 (0.84–1.17)
Oral cavity, pharynx	0.53 (0.24–1.17)	0.60 (0.23–1.59)	1.40 (0.41–4.72)	0.52 (0.24–1.13)	0.60 (0.23–1.61)	1.56 (0.43–5.65)
Peritoneum, omentum, mesentery	1.23 (0.33–4.57)	0.71 (0.19–2.65)	2.38 (0.29–19.60)	1.17 (0.31–4.42)	0.76 (0.21–2.70)	2.33 (0.31–17.80)
Soft tissue sarcoma	**2.38 (1.20–4.72)**	1.30 (0.78–2.19)	0.77 (0.40–1.50)	**2.36 (1.17–4.78)**	1.28 (0.76–2.17)	0.69 (0.34–1.38)
Melanoma of the skin	1.04 (0.62–1.74)	1.32 (0.79–2.22)	1.20 (0.57–2.55)	1.02 (0.61–1.70)	1.30 (0.78–2.17)	1.01 (0.46–2.18)
Contralateral breast^f^	1.17 (0.95–1.43)	0.81 (0.66–1.01)	**0.45 (0.35–0.56)**	1.22 (0.99–1.51)	0.82 (0.66–1.02)	**0.48 (0.38–0.60)**
Corpus uteri	1.36 (0.90–2.05)	1.10 (0.72–1.66)	1.03 (0.61–1.73)	1.38 (0.91–2.11)	1.10 (0.72–1.68)	1.06 (0.61–1.86)
Leukemia	1.68 (0.83–3.40)	0.72 (0.35–1.49)	0.81 (0.36–1.83)	1.78 (0.89–3.57)	0.75 (0.36–1.58)	0.83 (0.35–1.97)
Acute myeloid leukemia	3.38 (0.75–15.28)	1.45 (0.47–4.49)	1.00 (0.19–5.24)	3.47 (0.74–16.18)	1.35 (0.44–4.15)	0.92 (0.13–6.52)
Myelodysplastic syndrome^g^	2.31 (0.66–8.06)	2.84 (0.96–8.39)	1.36 (0.31–5.93)	2.09 (0.59–7.37)	**3.01 (1.01–8.94)**	1.08 (0.21–5.59)

Bold font indicates statistical significance

Abbreviations *HR*—Hazard Ratio, *CI*—Confidence interval, *KP*—Kaiser Permanente

^a^Results are presented for all second primary cancers, all second non-breast cancers, and select site-specific second cancers (overall SIRs ≥ 1.50)

^b^Fine and Gray regression models were used to estimate subdistribution hazard ratios accounting for death and other invasive cancer (site-specific analyses only) as a competing event

^c^Adjusted for age at first breast cancer (continuous)

^d^Adjusted for age at first breast cancer (continuous), diagnosis year for first breast cancer (< 1995, 1995- < 2000, 2000- < 2005, ≥ 2005), study site (KP Colorado, KP Northwest, KP Washington), and mutually adjusted for radiotherapy (yes, no), chemotherapy (yes, no), endocrine therapy (yes, no)

^e^Restricted to women diagnosed with a first estrogen receptor-positive breast cancer

^f^Excludes women with bilateral mastectomies (*n* = 1,042)

^g^Analyses for myelodysplastic syndrome are restricted to 2001–2017 (*n* = 12,746)

## Discussion

This study presents a comprehensive evaluation of second cancer risk among 16,004 breast cancer survivors diagnosed and treated within an integrated health care delivery system from 1990–2017. Despite advances in breast cancer treatment, our results demonstrate that breast cancer survivors continue to have an elevated second cancer risk, and risk varied by first breast cancer characteristics and treatment. This elevated risk is consistent with prior studies among patients with older treatment regimens [5–13]. Further, we observed that radiotherapy, chemotherapy, and endocrine therapy continue to be important treatment-related factors in second cancer risk. Our findings indicate that approximately 1 in 9 breast cancer patients developed a second cancer, 1 in 13 developed second non-breast cancer, and 1 in 30 developed a contralateral breast cancer by 10 years. Although we observed a decline in cumulative incidence for contralateral breast cancer, there was no decline in risk for second non-breast cancers. Importantly, these absolute risk estimates have remained similar to survivors diagnosed and treated prior to 2000 despite significant treatment advances [5]. Results from our study should heighten awareness for clinical surveillance and highlight the critical need to identify strategies to reduce second cancer risk.

Site-specific second cancers have been extensively studied in breast cancer survivors over the past four decades. Consistent with previous studies, we found elevated risk for malignancies of the contralateral breast [43–46], colon [5–10, 13, 18], pancreas [6, 8, 18], lung [5–8, 18, 47], oral cavity and pharynx [5, 6], uterine corpus [5–9, 11, 13, 14, 18], soft tissue [5–8, 10, 13, 14, 18], melanoma [5–10, 12, 13, 18], leukemia [5, 7, 8, 12–15, 18], and non-Hodgkin lymphoma [7, 18]. Elevated risk for these sites support shared genetic, hormonal, and/or lifestyle risk factors, and long-term effects of breast cancer treatment [5]. Although our results are inconsistent with prior studies suggesting an overall increased risk for ovarian [5–8, 10–15, 18] and thyroid [5, 6, 8, 18] cancers, we did observe higher risks for ovarian and thyroid cancer among younger women and ovarian cancer after ER-negative breast cancer, which may be indicative of genetic predisposition. In contrast to most prior studies, we also found elevated risk for peritoneal cancers and did not observe significantly elevated risks for malignancies of the esophagus [5–7, 18], bladder [7, 10, 18], or kidney [7, 13, 18]. The observed lower bladder cancer risk in our study may be related to differences in lifestyle factors among patients in the KP health care system (e.g., lower prevalence of smoking) compared with the general US population.

Our finding that breast cancer survivors have an over three-fold increased risk of contralateral breast cancer is likely related to hormonal, genetic, and other shared risk factors that predisposed women to develop the first breast cancer [5]. Although chemotherapy was associated with a statistically nonsignificant decreased risk of contralateral breast cancer in our study, several prior studies have found a significant risk reduction [16, 29, 30, 43, 48]. Few studies, however, have examined the effect of contemporary chemotherapy [16, 30], and further studies among patients treated in recent decades are warranted. In agreement with both clinical [32, 33] and observational studies [29–31], we found that endocrine therapy reduced contralateral breast cancer risk by over 50%. This finding underscores the importance to improve endocrine therapy initiation and adherence in women with ER-positive breast cancer.

The relative risk of soft tissue sarcoma in breast cancer survivors compared to that expected in the general population was over three-fold in our study. Soft tissue sarcoma risk was particularly elevated among younger women and associated with radiotherapy. The association between radiotherapy and soft tissue sarcoma has been well-reported among breast cancer patients treated with older treatment regimens [7, 12, 49–53]. However, a recent study in our cohort found that women treated with radiotherapy had an increased risk of developing thoracic soft tissue sarcomas, particularly angiosarcomas, but there was no association with prescribed dose, fractionation, or boost [54]. Future detailed studies examining modern treatment regimens and soft tissue sarcoma risk are warranted.

Risks of myelodysplastic syndrome and acute myeloid leukemia were also particularly elevated in our study, and we observed a three-fold increased risk of myelodysplastic syndrome associated with chemotherapy. Prior studies suggest that these elevated risks are likely related to chemotherapy [23, 55–58] and to a lesser extent radiotherapy [25, 57]. Although there have been multiple clinical trials and observational studies that have identified an increased risk of myelodysplastic syndrome and acute myeloid leukemia following chemotherapy, few have examined modern regimens [56, 58]. Notably, a recent study using SEER Medicare data found an increased use of known leukemogenic agents among breast cancer patients in recent calendar years [56].

Strengths of our study include a large cohort of breast cancer survivors within an integrated health care delivery system, which systematically captures aspects of care including cancer treatment and long-term follow-up. Radiotherapy and systemic treatments in SEER are for initial treatment only and even this is known to be under ascertained and classified as “no/unknown” for a large proportion of the population [35, 59]. Therefore, our study examines associations between treatment and second cancer risk that cannot be addressed with SEER data. Our results also reflect current treatment practices within a community-setting, and thus may have stronger external validity than clinical trials and subsequently may be more generalizable to the US breast cancer survivor population. However, our results may not be generalizable to survivors without health insurance and future studies examining the impact of health insurance status on second cancer risk are warranted. Finally, we restricted reference rates to first primary cancer incidence in the general US population. Prior studies have largely used first and higher order cancer incidence to calculate the expected rates which includes treatment-related cancers and thus may underestimate the risk of developing a second cancer after breast cancer. Restricting the reference rates to first primary cancers eliminates this downward bias.

Our study also has several limitations. Although we had comprehensive cancer and treatment information, our study lacked data on family history of cancer, as well as reproductive and genetic factors, including *BRCA1/2* and other mutation carrier status, and history of hysterectomy and oophorectomy. Additional studies examining the role of treatment with other shared etiologic factors, including genetic, lifestyle, and reproductive factors, are warranted to determine the primary and independent factors driving an increased risk of second cancer. It is possible that heightened medical surveillance may have contributed to elevated second cancer risks, particularly within the first 5 years after a breast cancer diagnosis. However, we found markedly elevated risks 5 + years after diagnosis which suggests that the late effects of treatment, as well as other shared etiologic factors, play an important role. Statistical power was limited to detect associations with smaller effect sizes for some site-specific second cancers, particularly among stratified models. Further, it is possible that some of the observed statistically significant associations in our study may be due to chance. Finally, our study included primarily non-Hispanic white women, and our results may not be generalizable to other races and ethnicities. Future studies among more diverse study populations are needed.

## Conclusions

This study found an elevated risk of second primary cancers in a large cohort of breast cancer survivors diagnosed and treated within an integrated health care delivery system. Our findings reflect contemporary US treatment practices and highlight the importance of heightened surveillance for second cancers among breast cancer survivors treated in recent decades. Observed second cancer risks, particularly for increased risk of soft tissue sarcoma after radiotherapy and myelodysplastic syndrome after chemotherapy, and decreased risk of breast cancer with endocrine therapy, warrant further investigation to mitigate carcinogenic effects and improve endocrine therapy initiation and adherence. Continued efforts are needed to identify prevention strategies to reduce second cancer risk in breast cancer survivors.

## Supplementary Information

**Additional file 1.** Risk of second primary cancer among women in the Kaiser Permanente Breast Cancer Survivors Cohort: Tables S1–S9 and Figures S1–S2.

## Data Availability

The data underlying this article will be shared on reasonable request to the corresponding author.

## Abbreviations

BMI: Body mass index
CI: Confidence interval
EMR: Electronic medical record
ER: Estrogen receptor
HER2: Human epidermal growth factor receptor 2
HR: Hazard ratio
KP: Kaiser Permanente
PR: Progesterone receptor
SEER: Surveillance, Epidemiology and End Results
SIR: Standardized incidence ratio
