# Supplementary material for: Risk of second primary cancer among women in the Kaiser Permanente Breast Cancer Survivors Cohort
Source: Breast Cancer Res. 2023 May 3;25:50. doi: 10.1186/s13058-023-01647-y (PMC10155401; doi:10.1186/s13058-023-01647-y)
Supplement: Supplementary file 1 — Additional file 1. Risk of second primary cancer among women in the Kaiser Permanente Breast Cancer Survivors Cohort: Tables S1–S9 and Figures S1–S2. [file 13058_2023_1647_MOESM1_ESM.docx]

| Table S1. Classification of second primary cancers based on ICD-O-3 site and histology codes | | |
| --- | --- | --- |
| Cancer types | ICD-O-3 Site | ICD-O-3 Histology^a^ |
| Oral cavity, pharynx | C000-C009, C019-C029, C079-C089, C040-C049, C030-C039, C050-C059, C060-C069, C110-C119, C090-C099, C100-C109, C129, C130-C139, C140, C142, C148 | Any histology excluding 9050-9055, 9590-9992 and soft tissue sarcoma histologies |
| Esophagus | C150-C159 |  |
| Stomach | C160-C169 |  |
| Colon | C180-C189, C260 |  |
| Rectum, rectosigmoid junction | C199, C209 |  |
| Liver, intrahepatic bile duct | C220-C221 |  |
| Pancreas | C250-C259 |  |
| Peritoneum, omentum, mesentery | C481-C482 |  |
| Lung, bronchus | C340-C349 |  |
| Soft tissue | Any site | 8587, 8710-8713, 8800-8806, 8810-8811, 8813-8815, 8820-8827, 8830-8836, 8840-8843, 8850-8858, 8860-8862, 8870, 8880-8881, 8890-8898, 8900-8905, 8910, 8912, 8920-8921, 8930-8931, 8933, 8932, 8934-8936, 8950-8951, 8964-8967, 8974-8979, 8980-8982, 8990-8991, 9040-9044, 9120-9125, 9130-9133, 9135, 9136, 9140 9150, 9160-9161, 9170-9175, 9180, 9210, 9220, 9231, 9240, 9251, 9252, 9260, 9363-9365, 9373, 9540-9571, 9491, 9580-9581 |
|  | C490-C499 | All other ICD-0-3 histology not otherwise specified above |
| Melanoma of the skin | C440-C449 | 8720-8790 |
| Breast | C500-C509 | Any histology excluding 9050-9055, 9590-9992, and soft tissue sarcoma histologies |
| Corpus uteri | C540-C549 |  |
| Ovary | C569 |  |
| Urinary bladder | C670-C679 |  |
| Kidney, renal pelvis | C649, C659 |  |
| Brain | C710-C719 | Any histology excluding 9050-9055, 9530-9539, 9590-9992, and soft tissue sarcoma histologies |
| Thyroid | C739 | Any histology excluding 9050-9055, 9590-9992, and soft tissue sarcoma histologies |
| Non-Hodgkin lymphoma | Any site | 9590-9597, 9670-9671, 9673, 9675, 9678-9680, 9684, 9687-9691, 9695, 9698-9702, 9705, 9708-9709, 9712, 9714-9719, 9724-9729, 9735, 9737-9738, 9811-9818, 9823, 9827, 9837 |
| Myeloma | Any site | 9731-9732, 9734 |
| Leukemia | C420, C421, C424 | 9811-9818, 9823, 9827, 9837 |
|  | Any site | 9733, 9742, 9800-9801, 9805-9809, 9820, 9823, 9826, , 9831-9836, 9840, 9860-9861, 9863, 9865-9867, 9869, 9870-9876, 9891, 9895-9897, 9898, 9910-9911, 9920, 9930, 9940, 9945-9946, 9948, 9963-9964 |
| Chronic lymphocytic  leukemia | C420, C421, C424 | 9823 |
| Acute myeloid leukemia | Any site | 9840, 9861, 9865-9867, 9869, 9871-9874, 9895-9897, 9898, 9910-9911, 9920 |
| Myelodysplastic syndrome^b^ | Any site | 9980, 9982-9989 |
| Miscellaneous | Any site | 9740-9741, 9750-9769, 9965-9967, 9970-9971, 9991-9992 |
|  | C420-C424, C760-C768, C770-C779, C809 | Any histology excluding 9050-9055, 9590-9992, and soft tissue sarcoma histologies |
| Abbreviations: ICD – International classification of diseases | | |
| ^a^ Soft tissue sarcomas were classified first and all other sites were classified excluding soft tissue sarcoma histology codes. Classification was based on the SEER site recode ICD-O-3/WHO 2008 definition available at: <https://seer.cancer.gov/siterecode/icdo3_dwhoheme/index.html> and based on an extended classification of the International Classification of Childhood Cancers, third edition, (ICCC-3)^1,2^ available at: <https://seer.cancer.gov/iccc/iccc3_ext.html> | | |
| ^b^ Diagnoses were restricted to 2001-2017 | | |

| **Table S2.** Cumulative incidence for site-specific second primary cancer among 16,004 women diagnosed with a first primary unilateral stage I-III breast cancer ^a^ | | |
| --- | --- | --- |
| Site-specific second primary cancer | 5 years | 10 years |
|  | % (95% CI) | % (95% CI) |
| All second primary cancers | 4.62 (4.27-4.99) | 10.85 (10.24-11.47) |
| All second primary non-breast cancers | 3.31 (3.02-3.63) | 7.53 (7.02-8.06) |
| Colon | 0.34 (0.25-0.45) | 0.88 (0.71-1.08) |
| Pancreas | 0.15 (0.09-0.23) | 0.33 (0.23-0.46) |
| Lung, bronchus | 0.54 (0.42-0.68) | 1.31 (1.11-1.55) |
| Soft tissue | 0.18 (0.12-0.27) | 0.43 (0.31-0.57) |
| Melanoma of the skin | 0.22 (0.15-0.31) | 0.44 (0.33-0.59) |
| Contralateral breast^b^ | 1.38 (1.19-1.60) | 3.40 (3.05-3.78) |
| Corpus uteri | 0.45 (0.35-0.58) | 0.90 (0.73-1.10) |
| Ovary | 0.09 (0.05-0.16) | 0.27 (0.18-0.39) |
| Non-Hodgkin Lymphoma | 0.19 (0.12-0.28) | 0.44 (0.32-0.60) |
| Leukemia | 0.13 (0.08-0.21) | 0.30 (0.21-0.43) |
| ^a^ Cumulative incidence for all second primary cancers, second non-breast cancers, and the ten most common second primary cancer sites were estimated accounting for the competing risk of death and other invasive cancers (site-specific analyses only) | | |
| ^b^ Women with bilateral mastectomies were excluded (n=1,042) | | |

| **Table S3.** SIRs for site-specific second primary cancer according to ER status of the first breast cancer among 16,004 women diagnosed with a first primary unilateral stage I-III breast cancer | | | | | | | |
| --- | --- | --- | --- | --- | --- | --- | --- |
|  | ER status of the first primary breast cancer | | | | | |  |
|  | Positive (n=12,764) | | | Negative (n=2,674) | | |  |
| Site-specific second primary cancer^a^ | O | E | SIR (95% CI) | O | E | SIR (95% CI) | P_heterogeneity_^b^ |
| Oral cavity, pharynx | 21 | 12.24 | **1.72 (1.06-2.62)** | <5 | -- | -- | -- |
| Esophagus | 5 | 4.44 | 1.13 (0.37-2.63) | <5 | -- | -- | -- |
| Stomach | 10 | 10.51 | 0.95 (0.46-1.75) | <5 | -- | -- | -- |
| Colon | 103 | 71.18 | **1.45 (1.18-1.75)** | 17 | 11.33 | 1.50 (0.87-2.40) | 0.89 |
| Rectum, rectosigmoid junction | 28 | 21.19 | 1.32 (0.88-1.91 | 6 | 3.61 | 1.66 (0.61-3.62) | 0.62 |
| Liver, intrahepatic bile duct | 12 | 9.09 | 1.32 (0.68-2.31) | <5 | -- | -- | -- |
| Pancreas | 35 | 28.21 | 1.24 (0.86-1.73) | 8 | 4.49 | 1.78 (0.77-3.51) | 0.37 |
| Peritoneum, omentum, mesentery | 10 | 2.37 | **4.21 (2.02-7.74)** | <5 | -- | -- | -- |
| Lung, bronchus | 151 | 124.01 | **1.22 (1.03-1.43)** | 36 | 19.98 | **1.80 (1.26-2.49)** | **0.04** |
| Soft tissue | 47 | 13.88 | **3.39 (2.49-4.50)** | 8 | 2.45 | **3.26 (1.41-6.43)** | 0.92 |
| Melanoma of the skin | 51 | 27.10 | **1.88 (1.40-2.47)** | 15 | 4.82 | **3.11 (1.74-5.13)** | 0.10 |
| Contralateral breast^c^ | 342 | 116.57 | **2.93 (2.63-3.26)** | 85 | 20.68 | **4.11 (3.28-5.08)** | **0.007** |
| Corpus uteri | 98 | 52.49 | **1.87 (1.52-2.28)** | 17 | 9.37 | **1.81 (1.06-2.90)** | 0.91 |
| Ovary | 30 | 26.27 | 1.14 (0.77-1.63) | 11 | 4.54 | **2.42 (1.21-4.33)** | **0.045** |
| Urinary bladder | 13 | 22.68 | **0.57 (0.31-0.98)** | <5 | -- | -- | -- |
| Kidney, renal pelvis | 24 | 19.69 | 1.22 (0.78-1.81) | 5 | 3.34 | 1.50 (0.49-3.49) | 0.68 |
| Brain | 16 | 8.77 | **1.82 (1.04-2.96)** | <5 | -- | -- | -- |
| Thyroid | 19 | 16.35 | 1.16 (0.70-1.81) | <5 | -- | -- | -- |
| Non-Hodgkin Lymphoma | 55 | 36.66 | **1.50 (1.13-1.95)** | 7 | 6.01 | 1.16 (0.47-2.40) | 0.51 |
| Myeloma | 16 | 11.81 | 1.36 (0.77-2.20) | <5 | -- | -- | -- |
| Leukemia | 32 | 22.00 | **1.45 (1.00-2.05)** | 6 | 3.53 | 1.70 (0.62-3.70) | 0.73 |
| Chronic Lymphocytic | 12 | 10.43 | 1.15 (0.59-2.01) | <5 | -- | -- | -- |
| Acute Myeloid Leukemia | 10 | 5.78 | 1.73 (0.83-3.18) | <5 | -- | -- | -- |
| Myelodysplastic syndrome^d^ | 15 | 4.42 | **3.39 (1.90-5.60)** | <5 | -- | -- | -- |
| Miscellaneous | 25 | 19.20 | 1.30 (0.84-1.92) | <5 | -- | -- | -- |
| Note: Bold font indicates statistical significance | | | | | | | |
| Abbreviations: SIRs – Standardized incidence ratios, O – Observed, E – Expected, ER – Estrogen receptor | | | | | | | |
| ^a^ Results with <5 events are omitted | | | | | | | |
| ^b^ Poisson regression with the observed number of cases as the outcome, the log of the expected number of cases as the offset, and the stratified factor as a covariate in the model was used to compare SIRs in stratified analyses.^3,4^ P-values for heterogeneity were based on the likelihood ratio statistic comparing model fit with and without the stratified factor | | | | | | | |
| ^c^ Women with bilateral mastectomies were excluded (n=1,042) | | | | | | | |
| ^d^ Myelodysplastic syndromes (MDS) diagnoses are restricted to 2001-2017 | | | | | | | |

| **Table S4.** SIRs for site-specific second primary cancer by age at first breast cancer among 16,004 women diagnosed with a first primary unilateral stage I-III breast cancer | | | | | | | | | | | | | |
| --- | --- | --- | --- | --- | --- | --- | --- | --- | --- | --- | --- | --- | --- |
|  |  | Age <45 (N=1,620) | | |  | Age <55 (N=5383) | | |  | Age ≥55 (N=10,621) | | |  |
| Site-specific second primary cancer^a^ |  | O | E | SIR (95% CI) |  | O | E | SIR (95% CI) |  | O | E | SIR (95% CI) | P_heterogeneity_^b,c^ |
| Oral cavity, pharynx |  | <5 | -- | -- |  | 8 | 3.10 | **2.58 (1.11-5.09)** |  | 17 | 12.04 | 1.41 (0.82-2.26) | 0.18 |
| Esophagus |  | <5 | -- | -- |  | <5 | -- | -- |  | 7 | 4.78 | 1.46 (0.59-3.01) | -- |
| Stomach |  | <5 | -- | -- |  | <5 | -- | -- |  | 10 | 11.38 | 0.88 (0.42-1.62) | -- |
| Colon |  | <5 | -- | -- |  | 13 | 10.16 | 1.28 (0.68-2.19) |  | 111 | 77.56 | **1.43 (1.18-1.72)** | 0.70 |
| Rectum, rectosigmoid junction |  | <5 | -- | -- |  | 5 | 5.17 | 0.97 (0.31-2.26) |  | 32 | 21.14 | **1.51 (1.04-2.14)** | 0.33 |
| Liver, intrahepatic bile duct |  | <5 | -- | -- |  | <5 | -- | -- |  | 13 | 9.48 | 1.37 (0.73-2.34) | -- |
| Pancreas |  | <5 | -- | -- |  | 6 | 3.96 | 1.51 (0.56-3.30) |  | 41 | 30.64 | 1.34 (0.96-1.82) | 0.78 |
| Peritoneum, omentum, mesentery |  | <5 | -- | -- |  | <5 | -- | -- |  | 8 | 2.46 | **3.25 (1.40-6.40)** | -- |
| Lung, bronchus |  | <5 | -- | -- |  | 25 | 19.54 | 1.28 (0.83-1.89) |  | 175 | 132.67 | **1.32 (1.13-1.53)** | 0.89 |
| Soft tissue |  | 9 | 0.81 | **11.06 (5.06-20.99)** |  | 18 | 3.95 | **4.55 (2.70-7.20)** |  | 39 | 13.24 | **2.95 (2.09-4.03)** | 0.14 |
| Melanoma of the skin |  | 7 | 2.41 | **2.90 (1.17-5.98)** |  | 28 | 9.74 | **2.88 (1.91-4.16)** |  | 38 | 23.69 | **1.60 (1.14-2.20)** | **0.02** |
| Contralateral breast^d^ |  | 47 | 7.70 | **6.10 (4.48-8.11)** |  | 145 | 37.74 | **3.84 (3.24-4.52)** |  | 304 | 107.00 | **2.84 (2.53-3.18)** | **0.003** |
| Corpus uteri |  | 5 | 2.72 | 1.84 (0.60-4.30) |  | 36 | 17.70 | **2.03 (1.42-2.82)** |  | 82 | 47.28 | **1.73 (1.38-2.15)** | 0.43 |
| Ovary |  | 5 | 1.38 | **3.63 (1.18-8.46)** |  | 7 | 7.26 | 0.96 (0.39-1.99) |  | 34 | 25.27 | 1.35 (0.93-1.88) | 0.41 |
| Urinary bladder |  | <5 | -- | -- |  | <5 | -- | -- |  | 16 | 24.50 | 0.65 (0.37-1.06) | -- |
| Kidney, renal pelvis |  | <5 | -- | -- |  | <5 | -- | -- |  | 27 | 19.51 | 1.38 (0.91-2.01) | -- |
| Brain |  | <5 | -- | -- |  | 5 | 2.25 | 2.22 (0.72-5.19) |  | 11 | 8.58 | 1.28 (0.64-2.29) | 0.33 |
| Thyroid |  | 8 | 2.63 | **3.04 (1.31-5.98)** |  | 14 | 8.94 | 1.57 (0.86-2.63) |  | 9 | 11.55 | 0.78 (0.36-1.48) | 0.10 |
| Non-Hodgkin Lymphoma |  | <5 | -- | -- |  | 9 | 7.27 | 1.24 (0.57-2.35) |  | 56 | 37.82 | **1.48 (1.12-1.92)** | 0.61 |
| Myeloma |  | <5 | -- | -- |  | <5 | -- | -- |  | 15 | 12.52 | 1.20 (0.67-1.98) | -- |
| Leukemia |  | <5 | -- | -- |  | 8 | 3.77 | 2.12 (0.92-4.18) |  | 33 | 23.22 | 1.42 (0.98-2.00) | 0.33 |
| Chronic Lymphocytic |  | <5 | -- | -- |  | <5 | -- | -- |  | 13 | 11.13 | 1.17 (0.62-2.00) | -- |
| Acute Myeloid Leukemia |  | <5 | -- | -- |  | <5 | -- | -- |  | 11 | 6.08 | 1.81 (0.90-3.24) | -- |
| Myelodysplastic syndrome^e^ |  | <5 | -- | -- |  | <5 | -- | -- |  | 14 | 4.97 | **2.81 (1.54-4.72)** | -- |
| Miscellaneous |  | <5 | -- | -- |  | <5 | -- | -- |  | 25 | 21.50 | 1.16 (0.75-1.72) | -- |
| Note: Bold font indicates statistical significance | | | | | | | | | | | | | |
| Abbreviations: SIRs – Standardized incidence ratios, O – Observed, E - Expected | | | | | | | | | | | | | |
| ^a^ Results with <5 events are omitted | | | | | | | | | | | | | |
| ^b^ Poisson regression with the observed number of cases as the outcome, the log of the expected number of cases as the offset, and the stratified factor as a covariate in the model was used to compare SIRs in stratified analyses.^3,4^ P-values for heterogeneity were based on the likelihood ratio statistic comparing model fit with and without the stratified factor | | | | | | | | | | | | | |
| ^c^ P-heterogeneity for SIRs stratified by age <55 years and ≥55 years at first breast cancer diagnosis | | | | | | | | | | | | | |
| ^d^ Women with bilateral mastectomies were excluded (n=1,042) | | | | | | | | | | | | | |
| ^e^ Myelodysplastic syndromes (MDS) diagnoses are restricted to 2001-2017 | | | | | | | | | | | | | |

| **Table S5.** SIRs for site-specific second primary cancer by receipt of radiotherapy for the first breast cancer among women diagnosed with a first primary unilateral stage I-III breast cancer | | | | | | | | | | | | | | |
| --- | --- | --- | --- | --- | --- | --- | --- | --- | --- | --- | --- | --- | --- | --- |
|  | 1-year survivors (n=16,004) | | | | | | | 5-year survivors (n=9,518)^a^ | | | | | | |
|  | Received radiation  (N=10638) | | | No/unknown radiation receipt (N=5366)^b^ | | |  | Received radiation (N=6,436)^c^ | | | No/unknown radiation receipt  (N=3,082)^d^ | | |  |
| Site-specific second primary cancer^e^ | O | E | SIR (95% CI) | O | E | SIR (95% CI) | P_het_^f,g^ | O | E | SIR (95% CI) | O | E | SIR (95% CI) | P_het_^f,g^ |
| Oral cavity | 13 | 10.08 | 1.29 (0.69-2.21) | 12 | 5.06 | **2.37 (1.23-4.15)** | 0.13 | 8 | 5.73 | **1.35 (1.10-1.63)** | 7 | 2.74 | **2.55 (1.03-5.26)** | 0.25 |
| Esophagus | 5 | 3.59 | 1.39 (0.45-3.25) | <5 | -- | -- | -- | <5 | -- | -- | <5 | -- | -- | -- |
| Stomach | 5 | 8.36 | 0.60 (0.19-1.40) | 7 | 4.66 | 1.50 (0.60-3.10) | 0.11 | <5 | -- | -- | <5 | -- | -- | -- |
| Colon | 73 | 56.68 | **1.29 (1.01-1.62)** | 51 | 31.04 | **1.64 (1.22-2.16)** | 0.19 | 47 | 33.48 | 1.40 (1.03-1.87) | 34 | 17.28 | **1.97 (1.36-2.75)** | 0.14 |
| Rectum | 21 | 17.27 | 1.22 (0.75-1.86) | 16 | 9.04 | **1.77 (1.01-2.88)** | 0.26 | 9 | 9.52 | 0.95 (0.43-1.79) | 10 | 4.75 | **2.10 (1.01-3.87)** | 0.08 |
| Liver | 10 | 7.42 | 1.35 (0.65-2.48) | <5 | -- | -- | -- | 5 | 4.51 | 1.11 (0.36-2.59) | <5 | -- | -- | -- |
| Pancreas | 29 | 22.68 | 1.28 (0.86-1.84) | 18 | 11.93 | 1.51 (0.89-2.39) | 0.58 | 19 | 14.06 | 1.35 (0.81-2.11) | 10 | 6.94 | 1.44 (0.69-2.65) | 0.87 |
| Peritoneum | 7 | 1.97 | **3.55 (1.43-7.31)** | <5 | -- | -- | -- | 6 | 1.16 | **5.19 (1.91-11.31)** | <5 | -- | -- | -- |
| Lung, bronchus | 121 | 101.24 | 1.20 (0.99-1.43) | 79 | 50.97 | **1.55 (1.23-1.93)** | 0.08 | 78 | 59.52 | **1.31 (1.04-1.64)** | 52 | 28.41 | **1.83 (1.37-2.40)** | 0.07 |
| Soft tissue | 47 | 11.57 | **4.06 (2.98-5.40)** | 10 | 5.62 | **1.78 (0.85-3.27)** | **0.01** | 29 | 6.57 | **4.41 (2.95-6.34)** | 5 | 3.06 | 1.64 (0.53-3.82) | **0.02** |
| Melanoma | 45 | 22.83 | **1.97 (1.44-2.64)** | 21 | 10.59 | **1.98 (1.23-3.03)** | 0.98 | 22 | 13.01 | **1.69 (1.06-2.56)** | 16 | 5.86 | **2.73 (1.56-4.44)** | 0.15 |
| Contralateral breast^h^ | 322 | 100.17 | **3.21 (2.87-3.58)** | 127 | 44.56 | **2.85 (2.38-3.39)** | 0.25 | 200 | 54.61 | **3.66 (3.17-4.20)** | 80 | 23.78 | **3.36 (2.66-4.18)** | 0.52 |
| Corpus uteri | 86 | 44.4 | **1.94 (1.55-2.39)** | 32 | 20.57 | **1.56 (1.06-2.20)** | 0.28 | 42 | 24.19 | **1.74 (1.25-2.35)** | 20 | 10.75 | **1.86 (1.14-2.87)** | 0.80 |
| Ovary | 29 | 21.75 | 1.33 (0.89-1.92) | 12 | 10.79 | 1.11 (0.57-1.94) | 0.59 | 21 | 12.08 | **1.74 (1.08-2.66)** | 9 | 5.76 | 1.56 (0.71-2.97) | 0.79 |
| Urinary bladder | 13 | 18.26 | 0.71 (0.38-1.22) | <5 | -- | -- | -- | 8 | 11.06 | 0.72 (0.31-1.43) | <5 | -- | -- | -- |
| Kidney, renal pelvis | 16 | 16.31 | 0.98 (0.56-1.59) | 15 | 7.94 | **1.89 (1.06-3.12)** | 0.07 | 12 | 9.52 | 1.26 (0.65-2.20) | 9 | 4.43 | 2.03 (0.93-3.86) | 0.29 |
| Brain | 10 | 7.25 | 1.38 (0.66-2.54) | 6 | 3.58 | 1.68 (0.62-3.65) | 0.71 | 9 | 4.13 | **2.18 (1.00-4.14)** | <5 | -- | -- | -- |
| Thyroid | 15 | 14.25 | 1.05 (0.59-1.74) | 8 | 6.25 | 1.28 (0.55-2.52) | 0.66 | 10 | 7.48 | 1.34 (0.64-2.46) | <5 | -- | -- | -- |
| NHL | 47 | 29.90 | **1.57 (1.16-2.09)** | 18 | 15.19 | 1.18 (0.70-1.87) | 0.30 | 29 | 17.6 | **1.65 (1.10-2.37)** | 13 | 8.49 | 1.53 (0.82-2.62) | 0.83 |
| Myeloma | 12 | 9.63 | 1.25 (0.64-2.18) | 5 | 4.90 | 1.02 (0.33-2.38) | 0.70 | 6 | 5.76 | 1.04 (0.38-2.27) | <5 | -- | -- | -- |
| Leukemia | 31 | 17.75 | **1.75 (1.19-2.48)** | 10 | 9.24 | 1.08 (0.52-1.99) | 0.17 | 18 | 10.75 | 1.68 (0.99-2.65) | 8 | 5.31 | 1.51 (0.65-2.97) | 0.80 |
| CLL | 8 | 8.43 | 0.95 (0.41-1.87) | 8 | 4.31 | 1.86 (0.80-3.66) | 0.18 | 6 | 5.20 | 1.15 (0.42-2.51) | 7 | 2.53 | **2.77 (1.11-5.70)** | 0.12 |
| AML | 13 | 4.68 | **2.78 (1.48-4.75)** | <5 | -- | -- | -- | 7 | 2.80 | **2.50 (1.01-5.15)** | <5 | -- | -- | -- |
| MDS^i^ | 14 | 3.47 | **4.04 (2.21-6.77)** | <5 | -- | -- | -- | <5 | -- | -- | <5 | -- | -- | -- |
| Miscellaneous | 20 | 15.03 | 1.33 (0.81-2.05) | 8 | 8.60 | 0.93 (0.40-1.83) | 0.38 | 11 | 9.16 | 1.20 (0.60-2.15) | 7 | 4.90 | 1.43 (0.57-2.94) | 0.72 |
| Note: Bold font indicates statistical significance | | | | | | | | | | | | | | |
| Abbreviations: SIRs – Standardized incidence ratios, O – Observed, E – Expected, CLL – Chronic lymphocytic leukemia, AML – Acute myeloid leukemia, MDS – Myelodysplastic syndrome | | | | | | | | | | | | | | |
| ^a^ Results restricted to 5-year survivors to account for the potential minimum latency period associated with radiation-associated cancers | | | | | | | | | | | | | | |
| ^b^ No/unknown receipt of radiotherapy is combined due to potential under ascertainment of radiotherapy in registry data (no radiotherapy: n=5,302; unknown radiotherapy: n=64) | | | | | | | | | | | | | | |
| ^c^ SIR (95% CI) for all sites excluding ipsilateral breast cancer=1.81 (1.67-1.96); SIR for all sites except any breast (95% CI)=1.48 (1.34-1.62) | | | | | | | | | | | | | | |
| ^d^ SIR (95% CI) for all sites excluding ipsilateral breast cancer=1.90 (1.70-2.12); SIR for all sites except any breast (95% CI)=1.69 (1.48-1.92) | | | | | | | | | | | | | | |
| ^e^ Results with <5 events are omitted | | | | | | | | | | | | | | |
| ^f^ Poisson regression with the observed number of cases as the outcome, the log of the expected number of cases as the offset, and the stratified factor as a covariate in the model was used to compare SIRs in stratified analyses.^3,4^ P-values for heterogeneity were based on the likelihood ratio statistic comparing model fit with and without the stratified factor | | | | | | | | | | | | | | |
| ^g^ P-values for heterogeneity for SIRs stratified by radiotherapy receipt (yes vs. no/unknown) | | | | | | | | | | | | | | |
| ^h^ Women with bilateral mastectomies were excluded (n=1,042) | | | | | | | | | | | | | | |
| ^i^ MDS diagnoses are restricted to 2001-2017 | | | | | | | | | | | | | | |

| **Table S6.** SIRs for site-specific second primary cancer by receipt of chemotherapy for the first breast cancer among 16,004 women diagnosed with a first primary unilateral stage I-III breast cancer | | | | | | | | |
| --- | --- | --- | --- | --- | --- | --- | --- | --- |
|  | Received chemotherapy (N=6786) | | |  | No chemotherapy receipt (N=9218) | | |  |
| Site-specific second primary cancer^a^ | O | E | SIR (95% CI) |  | O | E | SIR (95% CI) | P_heterogeneity_^b^ |
| Oral cavity, pharynx | 8 | 5.03 | 1.59 (0.69-3.14) |  | 17 | 10.11 | 1.68 (0.98-2.69) | 0.90 |
| Esophagus | <5 | -- | -- |  | 6 | 3.91 | 1.53 (0.56-3.34) | -- |
| Stomach | <5 | -- | -- |  | 8 | 9.53 | 0.84 (0.36-1.65) | -- |
| Colon | 27 | 23.39 | 1.15 (0.76-1.68) |  | 97 | 64.33 | **1.51 (1.22-1.84)** | 0.21 |
| Rectum, rectosigmoid junction | 11 | 8.36 | 1.32 (0.66-2.36) |  | 26 | 17.95 | 1.45 (0.95-2.12) | 0.79 |
| Liver, intrahepatic bile duct | <5 | -- | -- |  | 11 | 7.77 | 1.42 (0.71-2.53) | -- |
| Pancreas | 14 | 9.56 | 1.46 (0.80-2.46) |  | 33 | 25.05 | 1.32 (0.91-1.85) | 0.74 |
| Peritoneum, omentum, mesentery | <5 | -- | -- |  | 7 | 1.96 | **3.57 (1.44-7.35)** | -- |
| Lung, bronchus | 53 | 45.34 | 1.17 (0.88-1.53) |  | 147 | 106.87 | **1.38 (1.16-1.62)** | 0.30 |
| Soft tissue | 26 | 6.06 | **4.29 (2.80-6.29)** |  | 31 | 11.14 | **2.78 (1.89-3.95)** | 0.11 |
| Melanoma of the skin | 33 | 12.98 | **2.54 (1.75-3.57)** |  | 33 | 20.44 | **1.61 (1.11-2.27)** | 0.07 |
| Contralateral breast^c^ | 174 | 53.22 | **3.27 (2.80-3.79)** |  | 275 | 91.52 | **3.00 (2.66-3.38)** | 0.39 |
| Corpus uteri | 50 | 25.09 | **1.99 (1.48-2.63)** |  | 68 | 39.89 | **1.70 (1.32-2.16)** | 0.40 |
| Ovary | 12 | 11.14 | 1.08 (0.56-1.88) |  | 29 | 21.39 | 1.36 (0.91-1.95) | 0.50 |
| Urinary bladder | <5 | -- | -- |  | 14 | 19.98 | 0.70 (0.38-1.18) | -- |
| Kidney, renal pelvis | 10 | 8.13 | 1.23 (0.59-2.26) |  | 21 | 16.11 | 1.30 (0.81-1.99) | 0.88 |
| Brain | <5 | -- | -- |  | 13 | 7.17 | 1.81 (0.97-3.10) | -- |
| Thyroid | 12 | 9.72 | 1.23 (0.64-2.16) |  | 11 | 10.77 | 1.02 (0.51-1.83) | 0.65 |
| Non-Hodgkin Lymphoma | 17 | 13.89 | 1.22 (0.71-1.96) |  | 48 | 31.20 | **1.54 (1.13-2.04)** | 0.41 |
| Myeloma | <5 | -- | -- |  | 13 | 10.20 | 1.27 (0.68-2.18) | -- |
| Leukemia | 12 | 7.82 | 1.53 (0.79-2.68) |  | 29 | 19.17 | **1.51 (1.01-2.17)** | 0.97 |
| Chronic Lymphocytic | <5 | -- | -- |  | 13 | 9.07 | 1.43 (0.76-2.45) | -- |
| Acute Myeloid Leukemia | 7 | 2.08 | **3.37 (1.36-6.95)** |  | 8 | 5.04 | 1.59 (0.69-3.13) | 0.15 |
| Myelodysplastic syndrome^d^ | 10 | 1.39 | **7.20 (3.45-13.24)** |  | 7 | 3.84 | 1.82 (0.73-3.75) | **0.005** |
| Miscellaneous | 5 | 5.68 | 0.88 (0.29-2.05) |  | 23 | 17.95 | 1.28 (0.81-1.92) | 0.43 |
| Note: Bold font indicates statistical significance | | | | | | | | |
| Abbreviations: SIRs – Standardized incidence ratios, O – Observed, E - Expected | | | | | | | | |
| ^a^ Results with <5 events are omitted | | | | | | | | |
| ^b^ Poisson regression with the observed number of cases as the outcome, the log of the expected number of cases as the offset, and the stratified factor as a covariate in the model was used to compare SIRs in stratified analyses.^3,4^ P-values for heterogeneity were based on the likelihood ratio statistic comparing model fit with and without the stratified factor | | | | | | | | |
| ^c^ Women with bilateral mastectomies were excluded (n=1,042) | | | | | | | | |
| ^d^ Myelodysplastic syndromes diagnoses are restricted to 2001-2017 | | | | | | | | |

| **Table S7.** SIRs for site-specific second primary cancer by receipt of endocrine therapy for the first breast cancer among 12,746 women diagnosed with a first primary unilateral stage I-III ER-positive breast cancer | | | | | | | | |
| --- | --- | --- | --- | --- | --- | --- | --- | --- |
|  | Received endocrine therapy  (N=10526) | | |  | No endocrine therapy received  (N=2220) | | |  |
| Site-specific second primary cancer^a^ | O | E | SIR (95% CI) |  | O | E | SIR (95% CI) | P_heterogeneity_^b^ |
| Oral cavity, pharynx | 18 | 9.76 | **1.84 (1.09-2.91)** |  | <5 | -- | -- | -- |
| Esophagus | <5 | -- | -- |  | <5 | -- | -- | -- |
| Stomach | 9 | 8.14 | 1.11 (0.51-2.10) |  | <5 | -- | -- | -- |
| Colon | 77 | 55.16 | **1.40 (1.10-1.74)** |  | 26 | 16.02 | **1.62 (1.06-2.38)** | 0.51 |
| Rectum, rectosigmoid junction | 21 | 16.79 | 1.25 (0.77-1.91) |  | 7 | 4.40 | 1.59 (0.64-3.28) | 0.59 |
| Liver, intrahepatic bile duct | 10 | 7.21 | 1.39 (0.67-2.55) |  | <5 | -- | -- | -- |
| Pancreas | 28 | 22.01 | 1.27 (0.85-1.84) |  | 7 | 6.20 | 1.13 (0.45-2.33) | 0.78 |
| Peritoneum, omentum, mesentery | 9 | 1.92 | **4.70 (2.15-8.91)** |  | <5 | -- | -- | -- |
| Lung, bronchus | 117 | 98.33 | 1.19 (0.98-1.43) |  | 34 | 25.68 | 1.32 (0.92-1.85) | 0.59 |
| Soft tissue | 36 | 11.17 | **3.22 (2.26-4.46)** |  | 11 | 2.71 | **4.06 (2.03-7.27)** | 0.51 |
| Melanoma of the skin | 43 | 22.12 | **1.94 (1.41-2.62)** |  | 8 | 4.98 | 1.61 (0.69-3.16) | 0.61 |
| Contralateral breast^c^ | 226 | 94.55 | **2.39 (2.09-2.72)** |  | 116 | 22.02 | **5.27 (4.35-6.32)** | **<0.001** |
| Corpus uteri | 80 | 43.18 | **1.85 (1.47-2.31)** |  | 18 | 9.31 | **1.93 (1.15-3.05)** | 0.87 |
| Ovary | 24 | 21.04 | 1.14 (0.73-1.70) |  | 6 | 5.23 | 1.15 (0.42-2.50) | 0.99 |
| Urinary bladder | 10 | 17.77 | 0.56 (0.27-1.03) |  | <5 | -- | -- | -- |
| Kidney, renal pelvis | 22 | 15.83 | 1.39 (0.87-2.10) |  | <5 | -- | -- | -- |
| Brain | 12 | 7.03 | 1.71 (0.88-2.98) |  | <5 | -- | -- | -- |
| Thyroid | 15 | 13.78 | 1.09 (0.61-1.80) |  | <5 | -- | -- | -- |
| Non-Hodgkin lymphoma | 40 | 29.05 | 1.38 (0.98-1.87) |  | 15 | 7.61 | **1.97 (1.10-3.25)** | 0.25 |
| Myeloma | 12 | 9.33 | 1.29 (0.66-2.25) |  | 4 | 2.48 | 1.61 (0.44-4.13) | 0.70 |
| Leukemia | 24 | 17.25 | 1.39 (0.89-2.07) |  | 8 | 4.74 | 1.69 (0.73-3.32) | 0.64 |
| Chronic lymphocytic | 7 | 8.20 | 0.85 (0.34-1.76) |  | 5 | 2.22 | 2.25 (0.73-5.24) | 0.11 |
| Acute myeloid leukemia | 8 | 4.54 | 1.76 (0.76-3.47) |  | <5 | -- | -- | -- |
| Myelodysplastic syndrome^d^ | 13 | 3.40 | **3.82 (2.03-6.53)** |  | <5 | -- | -- | -- |
| Miscellaneous | 17 | 14.61 | 1.16 (0.68-1.86) |  | 8 | 4.59 | 1.74 (0.75-3.44) | 0.36 |
| Note: Bold font indicates statistical significance | | | | | | | | |
| Abbreviations: SIRs – Standardized incidence ratios, ER – Estrogen receptor, O – Observed, E - Expected | | | | | | | | |
| ^a^ Results with <5 events are omitted | | | | | | | | |
| ^b^ Poisson regression with the observed number of cases as the outcome, the log of the expected number of cases as the offset, and the stratified factor as a covariate in the model was used to compare SIRs in stratified analyses.^3,4^ P-values for heterogeneity were based on the likelihood ratio statistic comparing model fit with and without the stratified factor | | | | | | | | |
| ^c^ Women with bilateral mastectomies were excluded (n=1,042) | | | | | | | | |
| ^d^ Myelodysplastic syndromes diagnoses are restricted to 2001-2017 | | | | | | | | |

| **Table S8.** Multivariable-adjusted HRs and 95% CIs for breast cancer treatment and risk of developing select second primary cancers (overall SIR ≥ 1.50)^a,b,c^ | | | |
| --- | --- | --- | --- |
| Site-specific second primary cancer | Radiotherapy | Chemotherapy | Endocrine therapy^d^ |
| All second cancers | 1.05 (0.94-1.17) | 1.02 (0.90-1.14) | **0.78 (0.68-0.89)** |
| All second non-breast cancer | 0.98 (0.87-1.12) | 1.04 (0.90-1.20) | 0.97 (0.82-1.15) |
| Oral cavity, pharynx | 0.49 (0.22-1.09) | 0.70 (0.28-1.79) | 1.50 (0.43-5.20) |
| Peritoneum, omentum, mesentery | 1.06 (0.27-4.17) | 0.91 (0.20-4.13) | 2.21 (0.27-17.98) |
| Soft tissue | **2.23 (1.12-4.42)** | 1.46 (0.81-2.62) | 0.67 (0.33-1.37) |
| Melanoma of the skin | 0.97 (0.57-1.63) | 1.48 (0.86-2.54) | 0.96 (0.44-2.09) |
| Contralateral breast^e^ | 1.16 (0.94-1.42) | 0.98 (0.79-1.21) | **0.48 (0.38-0.60)** |
| Corpus uteri | 1.32 (0.88-1.99) | 1.24 (0.82-1.88) | 1.03 (0.61-1.76) |
| Leukemia | 1.67 (0.81-3.43) | 0.88 (0.42-1.87) | 0.82 (0.36-1.88) |
| Acute myeloid leukemia | 3.29 (0.74-14.71) | 1.54 (0.49-4.85) | 0.90 (0.18-4.51) |
| Myelodysplastic syndrome^f^ | 2.00 (0.57-7.02) | **3.14 (1.08-9.08)** | 1.07 (0.22-5.11) |
| Note: Bold font indicates statistical significance | | | |
| Abbreviations: HR - Hazard Ratio, CI – Confidence interval | | | |
| ^a^ Results are presented for second primary cancers, second non-breast cancers and select site-specific second cancers (overall SIRs ≥ 1.50) | | | |
| ^b^ HRs and 95% CIs were estimated using Cox proportional hazards regression where competing events are censored | | | |
| ^c^ Adjusted for age at first breast cancer (continuous), diagnosis year for first breast cancer (<1995, 1995-<2000, 2000-<2005, ≥2005), study site (KP Colorado, KP Northwest, KP Washington), and mutually adjusted for radiotherapy (yes, no), chemotherapy (yes, no), endocrine therapy (yes, no) | | | |
| ^d^ Restricted to women diagnosed with a first estrogen receptor-positive breast cancer (n=12,746) | | | |
| ^e^ Excludes women with bilateral mastectomies (n=1,042) | | | |
| ^f^ Myelodysplastic syndromes (MDS) diagnoses are restricted to 2001-2017 | | | |

| **Table S9.** Multivariable-adjusted HRs and 95% CIs for breast cancer treatment and risk of developing second primary cancer and non-breast cancer restricted to five-year breast cancer survivors, accounting for competing risks (n=9,518)^a,b,c^ | | | |
| --- | --- | --- | --- |
|  | Radiotherapy | Chemotherapy | Endocrine therapy^d^ |
| All second primary cancer | 0.99 (0.86-1.13) | **0.84 (0.72-0.98)** | 0.86 (0.73-1.03) |
| All second non-breast cancer | 0.92 (0.78-1.07) | 0.86 (0.72-1.04) | 1.07 (0.86-1.32) |
| Abbreviations: HR - Hazard Ratio, CI – Confidence interval | | | |
| ^a^ Results restricted to five-year survivors to account for the potential minimum latency period associated with radiation-associated cancers | | | |
| ^b^ Fine and Gray regression models were used to estimate subdistribution hazard ratios accounting for other invasive cancer and death as a competing event | | | |
| ^c^ Adjusted for age at first breast cancer (continuous), diagnosis year for first breast cancer (<1995, 1995-<2000, 2000-<2005, ≥2005), study site (KP Colorado, KP Northwest, KP Washington), and mutually adjusted for radiotherapy (yes, no), chemotherapy (yes, no), endocrine therapy (yes, no) | | | |
| ^d^ Restricted to women diagnosed with a first estrogen receptor-positive breast cancer | | | |

**Figure S1.** Identification of second primary cancer cases among women diagnosed with invasive unilateral breast cancer between 1990-2016 (followed through 2017) in the Kaiser Permanente Breast Cancer Survivors’ Cohort. Women diagnosed as having invasive breast cancer at Kaiser Permanente (KP) Northwest Center for Health Research (Oregon) (1990-2008), KP Institute for Health Research (Colorado) (1994-2014) or KP Washington Health Research Institute (Washington) (1990-2016) were followed until the first of the following events: second cancer (invasive cancer or in situ breast cancer), death, end of KP eligibility, or end of the study (December 31, 2010, for KP Northwest; December 31, 2015 for KP Colorado; December 31, 2017 for KP Washington).

**
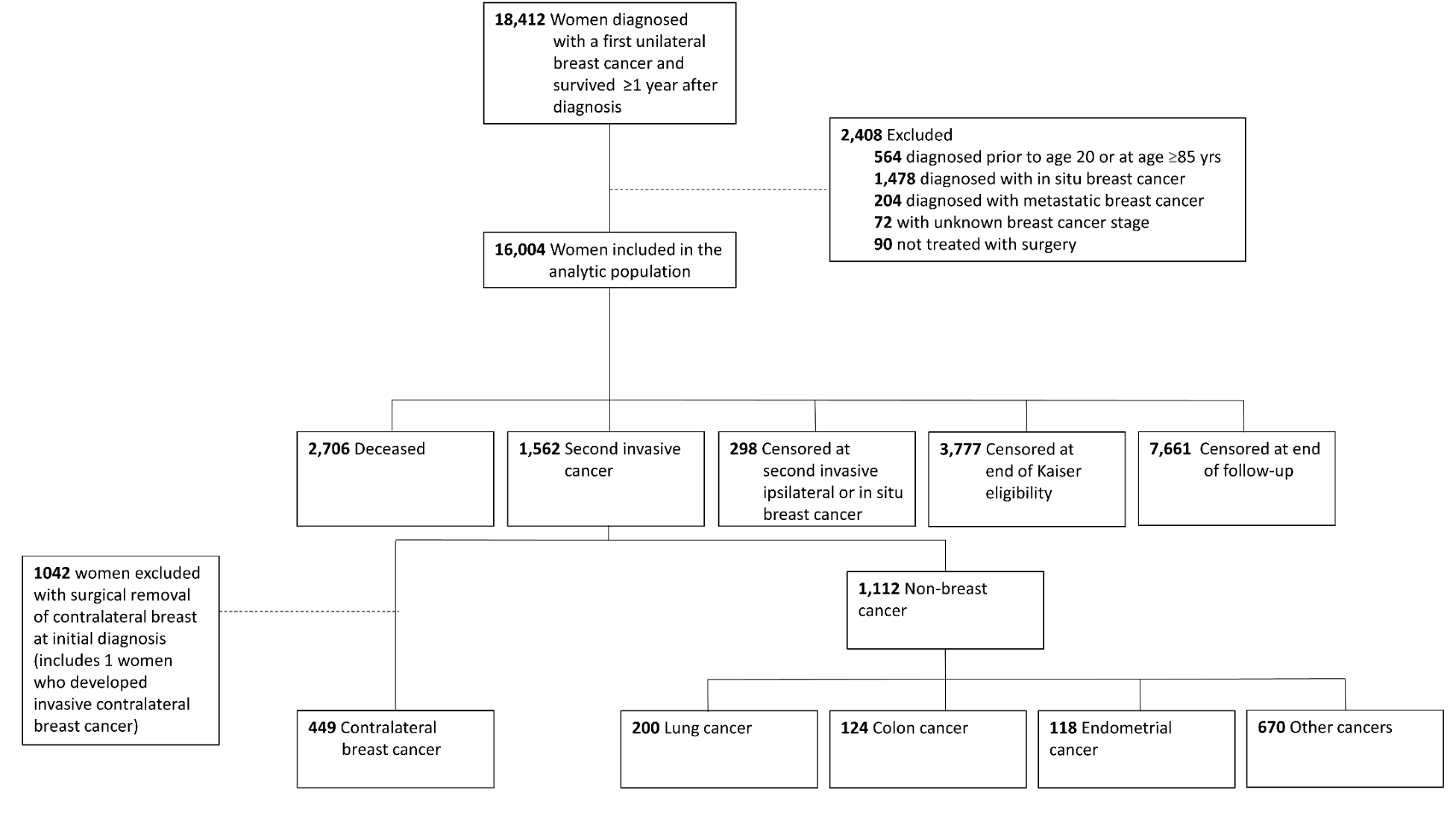
**

**Figure S2.** Cumulative incidence for all second primary cancer, second non-breast cancer, and contralateral breast cancer according to year of first breast cancer diagnosis among 16,004 women diagnosed with a first primary unilateral stage I-III breast cancer

**
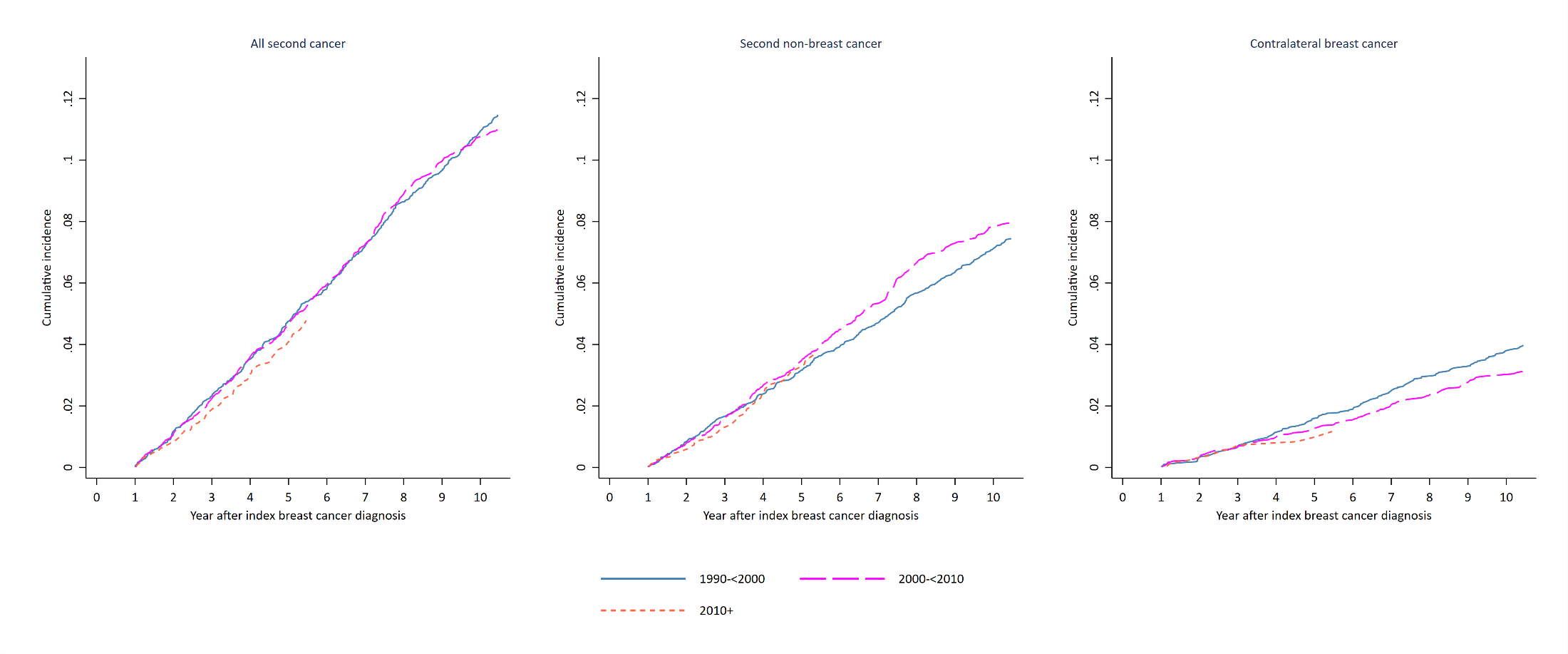
**

**Supplementary References**

1. Kleinerman RA, Schonfeld SJ, Sigel BS, Wong-Siegel JR, Gilbert ES, Abramson DH, S et al. Bone and soft‐tissue sarcoma risk in long‐term survivors of hereditary retinoblastoma treated with radiation. J Clin Oncol. 2019;37(35):3436-45.

2. Steliarova-Foucher E, Stiller C, Lacour B, Kaatsch P. International classification of childhood cancer, 3rd edition. 2005;103(7):1457-67.

3. Yasui Y, Liu Y, Neglia JP, Friedman DL, Bhatia S, Meadows AT, et al. A methodological issue in the analysis of second-primary cancer incidence in long-term survivors of childhood cancers. Am J Epidemiol. 2003;158(11):1108-13

4. Rostgaard K. Methods for stratification of person-time and events – a prerequisite for poisson regression and sir estimation. Epidemiol Perspect Innov. 2008;5(1):7.
